# Deletion of integrin α7 subunit does not aggravate the phenotype of laminin α2 chain-deficient mice

**DOI:** 10.1038/srep13916

**Published:** 2015-09-10

**Authors:** Kinga I. Gawlik, Madeleine Durbeej

**Affiliations:** 1 Department of Experimental Medical Science, Muscle Biology Unit, Lund University, Sweden.

## Abstract

Laminin-211 is a major constituent of the skeletal muscle basement membrane, exerting its biological functions by binding to cell surface receptors integrin α7β1 and dystroglycan (the latter is part of the dystrophin-glycoprotein complex). The importance of these molecules for normal muscle function is underscored by the fact that their respective deficiency leads to different forms of muscular dystrophy with different severity in humans and animal models. We recently demonstrated that laminin α2 chain and members of the dystrophin-glycoprotein complex have overlapping but non-redundant roles despite being part of the same adhesion complex. To analyse whether laminin-211 and integrin α7 subunit have non-redundant functions we generated mice deficient in laminin α2 chain and integrin α7 subunit (*dy*^*3K*^/*itga7*). We show that lack of both molecules did not exacerbate the severe phenotype of laminin α2-chain deficient animals. They displayed the same weight, survival and dystrophic pattern of muscle biopsy, with similar degree of inflammation and fibrosis. These data suggest that laminin-211 and integrin α7β1 have intersecting roles in skeletal muscle.

The mechanical stability and function of skeletal muscle are largely dependent on the association of the extracellular matrix with the muscle cell membrane and the intracellular cytoskeleton. The significance of this cell-matrix continuity is highlighted in humans as mutations in the genes encoding extracellular matrix, cell surface receptors or cytoskeleton-interacting proteins cause various types of muscular dystrophy^1^. A central element of the extracellular matrix-muscle fiber linkage is laminin-211, a heterotrimeric cell adhesion molecule (composed of α2, β1 and γ1 subunit) that strengthens sarcolemmal stability, protects muscles from damage, controls myofiber survival and regeneration and regulates muscle function^2^,^3^,^4^,^5^,^6^. The deficiency of laminin α2 chain leads to a severe form of congenital muscular dystrophy (type 1A, referred to as MDC1A)^7^,^8^,^9^. Moreover, complete deficiency of laminin α2 chain in mice (*dy*^3*K*^*/dy*^*3K*^ animals used in this study) results in the most severe phenotype among mouse models lacking proteins of cell adhesion complexes^2^,^10^,^11^,^12^.

Integrin α7β1 is one of the major cell surface receptors that binds laminin-211 in the musculoskeletal system^13^,^14^. Integrin α7β1 is localized both at the sarcolemma and enriched at the neuromuscular and myotendinous junctions^15^. In addition to providing anchorage and structural framework integrin α7β1 mediates signalling transduction cues^16^,^17^,^18^. This dual role has been suggested to be an engine for numerous processes: muscle development (myoblast migration, fusion, differentiation *in vitro*), survival, growth, regeneration and force generation^5^,^15^,^18^,^19^,^20^,^21^,^22^. Yet, skeletal muscle develops normally in integrin α7-null mice. Additionally, these mice are presented with a mild form of muscular dystrophy that mostly affects myotendinous junction^23^,^24^. Similarly, the loss of integrin α7 subunit in humans causes an extremely rare form of congenital myopathy^25^,^26^.

Another protein complex that interacts with laminin-211 and comprises the molecular link between extracellular matrix and cytoskeleton is the dystrophin-glycoprotein complex (DGC). Numerous research investigations have addressed the role of DGC in preservation of the structural and functional integrity of skeletal muscle^5^,^9^,^27^,^28^.

In spite of the relatively mild phenotype of integrin α7-deficient muscle, several studies in mice and humans indicated that integrin α7 subunit could play a significant role in modifying the disease progression in muscular dystrophies involving the absence of DGC components (dystrophin- and γ-sarcoglycan-deficient muscular dystrophies)^29^,^30^,^31^,^32^,^33^,^34^. These studies suggested that integrin α7β1 and the DGC could have redundant functions. Yet, the separate roles for these two complexes are also evident^5^,^35^. Even the interaplay between laminin α2 chain and integrin α7β1 seems to be complex and its role in skeletal muscle has not been fully deciphered. Reduced expression of integrin α7 subunit^20^,^29^,^36^ and its aberrant deposition at the sarcolemma^37^ accompany the laminin α2 chain loss. Consequently, overexpression of integrin α7 subunit in laminin α2 chain-deficient mice improves muscle pathology^38^. Nonetheless, the phenotypes of integrin α7 and laminin α2 chain-deficient mice and patients are markedly different^9^,^11^,^23^.

We have recently demonstrated that the absence of laminin α2 chain in dystrophin or β-sarcoglycan deficiency drastically aggravates the phenotype of *mdx* and *Sgcb* mice, respectively^12^. Our results pointed out that laminin α2 chain, dystrophin, and β-sarcoglycan have complementary, but non-redundant roles in spite of being part of the same adhesion complex. In the current study we generated laminin α2 chain-integrin α7 chain double knockout mice (*dy*^*3K*^/*itga7*) in order to establish the relationship between these molecules independently of the DGC and to determine if the main role of integrin α7β1 is to mediate laminin-211 interactions in muscle.

## Results

### Expression profile of integrins in laminin α2 chain- and integrin α7-deficient muscles

**Figure 1:**
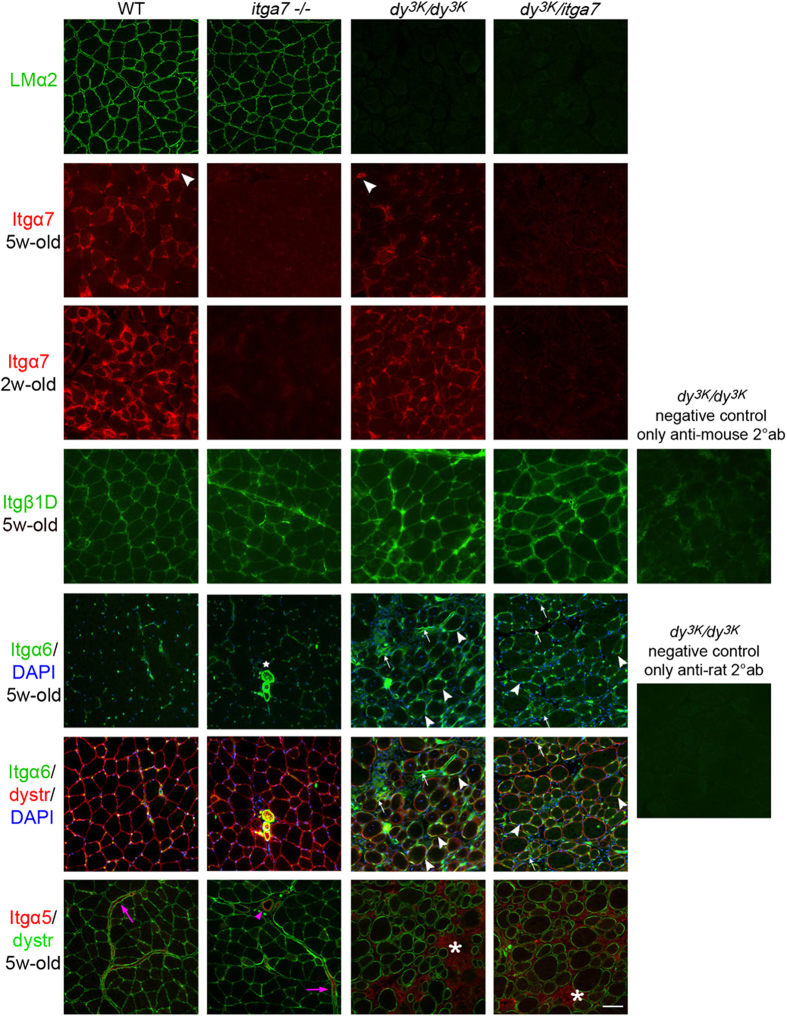
Immunostaining with antibodies against laminin α2 chain (green, LMα2) and integrin α7 chain (red, Itgα7) confirms the complete absence of both proteins in *dy*^*3K*^/*itga7* double knockout muscles. Expression of integrin α7 subunit is reduced at the sarcolemma of 5-week-old *dy*^*3K*^*/dy*^*3K*^ laminin α2 chain-deficient muscles. However, integrin α7 chain is present at the sarcolemma of the majority of 2-week-old *dy*^*3K*^*/dy*^*3K*^ muscle fibers. White arrowheads show maintained expression of integrin α7 in vessels. Laminin α2 chain is not reduced in integrin α7 knockout muscles. Expression of integrin β1D subunit (Itgβ1D, green) is maintained in muscles from all three mutants. Integrin α6 chain (Itgα6, green) is expressed in big and small vessels and peripheral nerves (white star), but not at the sarcolemma of normal muscle and *itga7*-null muscle. In addition to normal expression in vessels and nerves, integrin α6 subunit is present in muscle cell membranes in small regenerating *dy*^*3K*^*/dy*^*3K*^ and *dy*^*3K*^/*itga7* muscle fibers (white arrows) and weakly expressed at the sarcolemma in some bigger muscle fibers (white arrowheads). Sections were co-stained with the antibody against dystrophin (dystr, red) and DAPI (blue). Integrin α5 subunit (Itgα5, red) was massively upregulated in fibrotic lesions and/or sites of inflammation in laminin α2 chain-null mice and laminin α2 chain/integrin α7 double knockout animals (white asterisk), but was not found to be deposited at the sarcolemma. In normal muscle and in integrin α7-deficient muscle integrin α5 chain is expressed only in bigger vessels (pink arrowheads) and in interstitial connective tissue (pink arrows). Muscle sections were co-stained with dystrophin antibody (dystr, green). Scale bars, 40 μm.

*Dy*^*3K*^*/dy*^*3K*^ mice exhibit very severe muscular dystrophy^10^,^12^. In order to determine if the deletion of integrin α7 subunit exacerbates the phenotype of laminin α2 chain-deficient dystrophic mice, we generated mice lacking both molecules (*dy*^*3K*^/*itga7*) (by series of heterozygous breeding, see materials and methods). The genotype was confirmed by PCR (data not shown) and immunofluorescence (Fig. 1). In accordance with previous studies, laminin α2 chain was normally expressed in the absence of integrin α7 subunit^36^ (Fig. 1). Integrin α7 chain, on the other hand, was reduced in the *dy*^*3K*^*/dy*^*3K*^ laminin α2 chain-deficient muscle^37^ (Fig. 1). Yet, compared with *itga7* knockout mice and double knockout muscles, integrin α7 subunit was not completely absent from the sarcolemma of laminin α2 chain-deficient mice and it was also present in vessels (Fig. 1). Furthermore, the expression of integrin α7 subunit in younger (2-week-old) *dy*^*3K*^*/dy*^*3K*^ muscles was maintained at the sarcolemma (Fig. 1). Taken together, these results clearly distinguish *dy*^*3K*^*/dy*^*3K*^ muscles from *dy*^*3K*^/*itga7* double knockout muscles in terms of integrin α7 chain expression. Additionally, integrin β1 subunit, which forms a dimer with integrin α7 chain in healthy muscle, was expressed in all three knockout models^37^ (Fig. 1). Hence, we investigated the expression of integrin α subunits that could potentially dimerize with integrin β1 chain at the sarcolemma. Integrin α6β1 is a laminin-211 receptor and integrin α6 subunit has been demonstrated to be upregulated in mice with partial deficiency of laminin α2 chain (*dy/dy* mouse model)^39^. Integrin α5 chain is another candidate to form a dimer with integrin β1 subunit in muscle, as it has been shown to contribute to maintenance of normal muscle morphology^40^. We found moderate upregulation of integrin α6 subunit in *dy*^3*K*^*/dy*^3*K*^ and *dy*^*3K*^/*itga7* muscles compared to wild-type and *itga7* muscles (Fig. 1). Integrin α6 was mostly present in smaller regenerating fibers (Fig. 1, arrows), but it was also occasionally found in a few bigger muscle fibers, though its expression was rather weak and not continuous throughout the sarcolemma (Fig. 1, arrowheads). Integrin α5 chain was expressed only in large vessels and in streaks of interstitial connective tissue in wild-type and integrin α7-null muscle (Fig. 1). It was not deposited at the sarcolemma of *dy*^*3K*^*/dy*^*3K*^ and *dy*^*3K*^/*itga7* muscles, but instead it was massively upregulated at the interstitial space within muscle fibers (Fig. 1), suggesting a role for integrin α5 in inflammation and fibrosis.

Integrin α7β1 binds also laminin α4 and α5 chains^14^. Those laminin chains are upregulated upon laminin α2 chain loss in skeletal muscle^41^. Accordingly, we detected their upregulation in the extrasynaptic basement membranes in *dy*^*3K*^*/dy*^*3K*^ and *dy*^*3K*^/*itga7* muscles (Supplementary Fig. S1). Additionally, we found laminin α4 and laminin α5 chains to be weakly expressed at the myotendinous junction (MTJ) in wild-type mice. Their expression was preserved (laminin α5) or increased (laminin α4) at this site in all three analysed mutants, which could partially explain maintained expression of integrin α7β1 at the MTJ in *dy*^*3K*^*/dy*^*3K*^ mice and integrin β1 presence in double knockout mice (Supplementary Fig. S1). Integrin α5 and α6 have also been found to be moderately expressed at the MTJ in all three knockout mice (Supplementary Fig. S1).

Taken together, the expression pattern of integrin α5β1, α6β1, laminin α4 and α5 chains is the same between *dy*^*3K*^*/dy*^*3K*^ and *dy*^*3K*^/*itga7* muscle, indicating no additional compensatory mechanisms in double knockout mice.

### Overall phenotype of *dy* ^3*K*^/*itga7* double knockout mice is not aggravated compared with *dy* ^*3K*^ */dy* ^*3K*^ single knockouts

**Figure 2:**
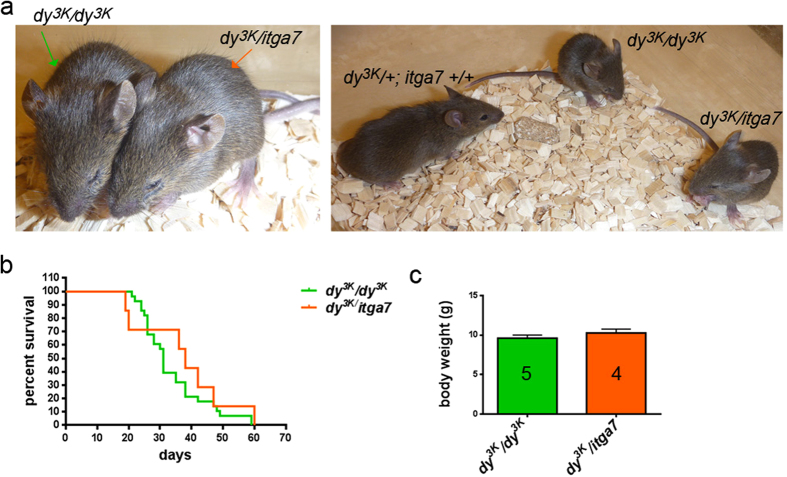
General health of laminin α2 chain/integrin α7-deficient mice is not worsened compared to severely affected laminin α2 chain single knockout animals. (**a**) Photos of 4-week-old *dy*^*3K*^*/dy*^*3K*^ and *dy*^*3K*^/*itga7* mice (left picture) with a normal littermate (right picture). Both mutants are severely emaciated and display dystrophic phenotype, with severe loss of muscle tissue. (**b**) Lifespan of *dy*^*3K*^/*itga7* is not shortened compared to *dy*^*3K*^*/dy*^*3K*^ mice (survival curves are not significantly different, p = 0.2331, log-rank Cox-Mantel test). Note that much fewer double knockout mice were taken under consideration due to partial embryonic lethality of *itga7* mice. (**c**) The whole body weight is not significantly different between *dy*^*3K*^*/dy*^*3K*^ and *dy*^*3K*^/*itga7* mice (p = 0.3175, Mann-Whitney test). The numbers of animals used are indicated in the graph.

Approximately 60% of *itga7* null-mice are embryonic lethal due to cerebrovascular defects^42^. Thus, the number of *dy*^*3K*^/*itga7* mice used in this study was limited. Integrin α7-deficient animals that survive embryonic development have near normal life span, weight and are fertile. Double knockout mice that were born did not die earlier than *dy*^*3K*^*/dy*^*3K*^ mice and both mutants lived up to 7–8 weeks after birth (Fig. 2b) (*dy*^*3K*^*/dy*^*3K*^ mice usually die at around 3–4 weeks of age, but we observed increased survival on the B6;129-*Itga7*^*tm1Burk*^/J background). Likewise, the overall health of *dy*^*3K*^/*itga7* mice was not worsened compared to *dy*^*3K*^*/dy*^*3K*^ animals. They displayed comparable growth retardation, muscle wasting, tremor, scoliosis and reduced eagerness to move (Fig. 2a). Accordingly, the weight of *dy*^*3K*^/*itga7* mice was not significantly different from single knockout *dy*^*3K*^*/dy*^*3K*^ animals (Fig. 2c).

### Dystrophic features of laminin α2 chain-deficient muscle are not dramatically changed upon integrin α7 deletion

**Figure 3:**
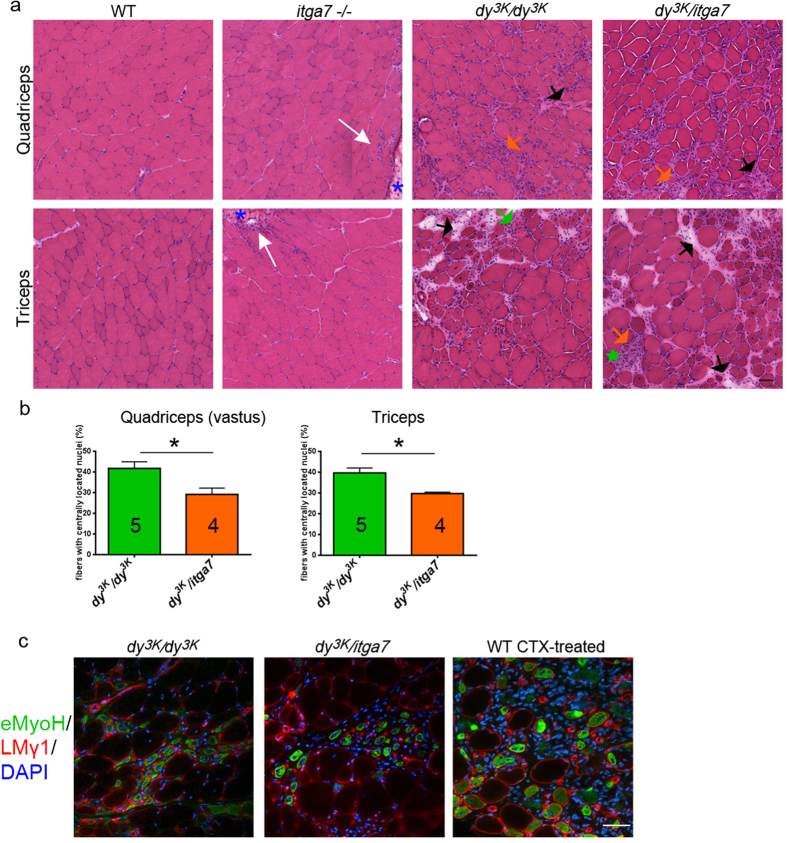
Muscular dystrophy hallmarks are not exacerbated in limb muscles from *dy*^*3K*^/*itga7* mice. (**a**) Hematoxylin and eosin staining of quadriceps and triceps muscles from 5–7-week-old mice shows mild myopathy (white arrows) at the myotendinous junctions (blue asterisk) in *itga7* −/− mice and severe muscular dystrophy with robust inflammation (orange arrows), muscle damage (single degenerating fibers or areas with a group of damaged fibers/fiber debris are indicated with green arrow or green star, respectively), muscle regeneration (fibers with centrally located nucleus) and connective tissue build-up (black arrows) in *dy*^*3K*^*/dy*^*3K*^ and *dy*^*3K*^/*itga7* animals. (**b**) Quantification of centrally nucleated fibers shows significant decrease in number of regenerating fibers in *dy*^*3K*^/*itga7* quadriceps (vastus intermedius) and triceps compared with *dy*^*3K*^*/dy*^*3K*^ corresponding muscles (p = 0.0317; p = 0.0159, respectively; Mann-Whitney test). The numbers of animals used are indicated in the graph. (**c**) Embryonic myosin heavy chain staining (eMyoH, green) reveals ongoing regeneration in both *dy*^*3K*^*/dy*^*3K*^ and *dy*^*3K*^/*itga7* muscles. Sections were costained with DAPI (blue) and laminin γ1 (LMγ1, red) to visualize nuclei and delineate muscle fibers, respectively. Wild-type muscle treated with cardiotoxin (CTX) is shown as a positive control for embryonic myosin heavy chain staining. Scale bars, 30 μm.

Laminin α2 chain-deficient muscle displays multiple defects. Cell death and degeneration/regeneration cycles are concomitant with massive infiltration of inflammatory cells and subsequent robust production of extracellular matrix components^2^ (Fig. 3a). These pathogenic processes result in severe muscle wasting and loss of muscle function. Integrin α7-null mice, on the other hand, develop mild myopathy with defects concentrated around the myotendinous junction – with its disrupted structure and function as well as with myofiber necrosis, muscle regeneration and inflammation around the myotendionous junctions^23^,^24^ (Fig. 3a).

Histological analysis of limb muscle sections from 5–7-week-old double knockout *dy*^*3K*^/*itga7* mice did not reveal additional changes in muscle morphology compared to the severe defects seen in *dy*^*3K*^*/dy*^*3K*^ muscles (Fig. 3a). Muscle fascicles were equally severely disrupted in both mutants, with large areas of focal apoptosis/necrosis and mononuclear cells infiltrates (Fig. 3a). Regenerating fibers with centrally located nuclei and loose atrophic fibers were also present. Nevertheless, quantification of centrally nucleated muscle cells showed decreased number of regenerating fibers in *dy*^*3K*^/*itga7* muscles (triceps and quadriceps) compared to *dy*^*3K*^*/dy*^*3K*^ muscles (Fig. 3b). We next estimated the regenerative capacity of *dy*^*3K*^/*itga7* muscle by immunostaining using the antibody against the embryonic myosin heavy chain. Newly regenerating fibers expressing embryonic form of myosin heavy chain were present in both *dy*^*3K*^*/dy*^*3K*^ and *dy*^*3K*^/*itga7* muscles (Fig. 3c), indicating that muscle regeneration is not exhausted in double knockout mice. Yet, the regenerative capacity could still be altered/slowed down in the absence of integrin α7 subunit, as indicated by decreased numbers of regenerating cells.

**Figure 4:**
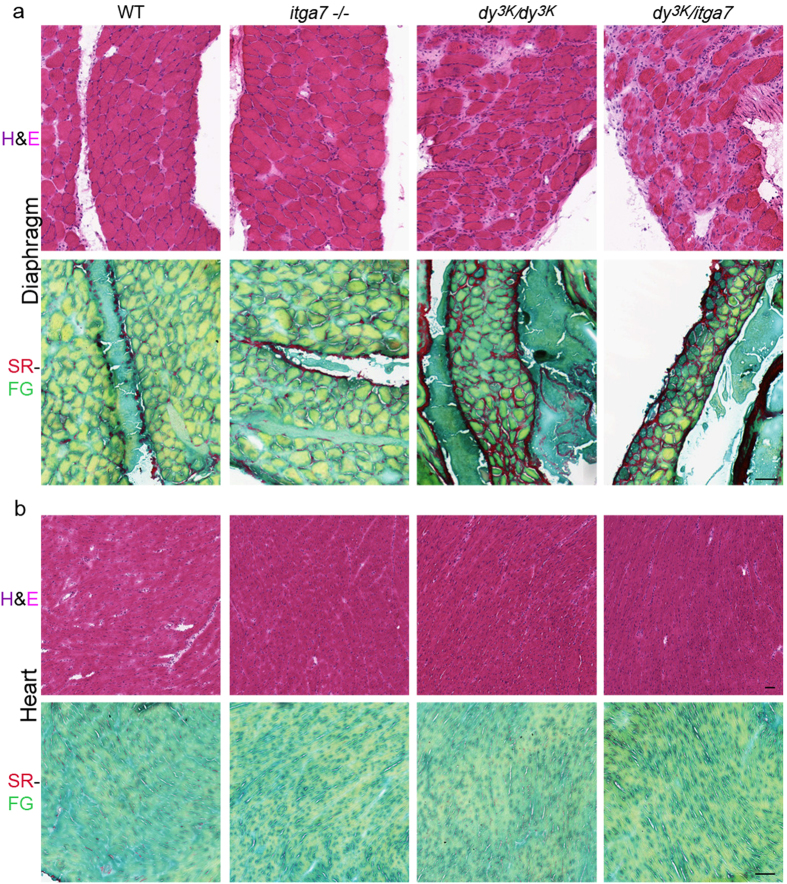
No differences in the phenotype of diaphragm and heart between *dy*^*3K*^*/dy*^*3K*^ and *dy*^*3K*^/*itga7* mice. (**a**) Hematoxylin and eosin staining (H&E) and Sirius red/fast green staining (SR-FG) demonstrate dystrophic features in diaphragm from *dy*^*3K*^*/dy*^*3K*^ and *dy*^*3K*^/*itga7* mice. Loss of muscle fibers, inflammation and connective tissue build up are evident (H&E) in these mutants. Presence of fibrosis was confirmed by Sirius red staining (collagen deposition, dark pink). No dystrophic changes were observed in *itga7* −/− diaphragm. (**b**) Hematoxylin and eosin staining (H&E) and Sirius red/fast green staining (SR-FG) do not show cardiomyopathy features in hearts from single and double knockout mice. Scale bars, 50 μm.

A general examination of the *dy*^*3K*^/*itga7* diaphragm did not reveal a dramatic aggravation of the phenotype either (Fig. 4a). Both *dy*^*3K*^*/dy*^*3K*^ and *dy*^*3K*^/*itga7* diaphragm showed dystrophic features (Fig. 4a, hematoxylin&eosin staining) and rich collagen deposition (Fig. 4a, Sirius red/fast green staining). As it has been shown before, heart muscle from *dy*^*3K*^*/dy*^*3K*^ mice was not affected^35^ (Fig. 4b), despite laminin α2 chain being abundantly deposited in heart basement membranes. Integrin α7 subunit is also strongly expressed in heart^43^. However, in addition to laminin α2 chain loss, the absence of integrin α7 subunit in *dy*^*3K*^/*itga7* heart did not result in appearance of cardiomyopathy features and visibly increased collagen content (Fig. 4b).

**Figure 5:**
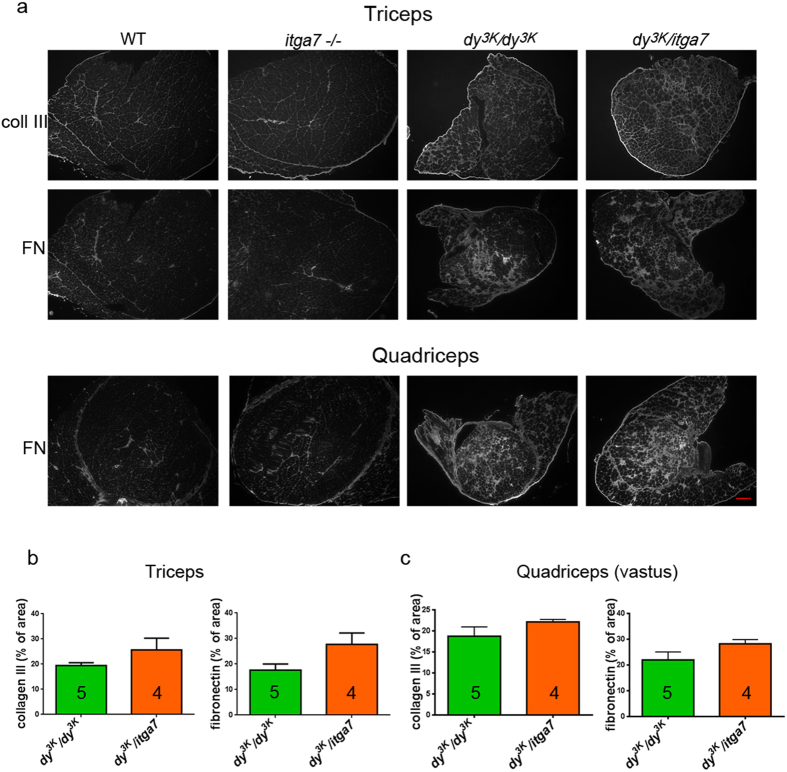
Fibrotic lesions are equally abundant in muscles from *dy*^*3K*^*/dy*^*3K*^ and *dy*^*3K*^/*itga7* mice. (**a**) Immunofluorescence with antibodies against collagen III (coll III) and fibronectin (FN) demonstrate extensive production of fibrotic proteins in *dy*^*3K*^*/dy*^*3K*^ and *dy*^*3K*^/*itga7* muscles. (**b**) Fibronectin and collagen III deposition was not statistically different between these two groups in triceps (p = 0.0635 and p = 0.0635, respectively; Mann-Whitney) and quadriceps (vastus intermedius) (p = 0.1905 and p = 0.1905, respectively; Mann-Whitney). Yet, the p values for triceps muscle could indicate a trend for slightly larger areas of collagen III and fibronectin in *dy*^*3K*^/*itga7* triceps in comparison with *dy*^*3K*^*/dy*^*3K*^ specimens. The numbers of animals used are indicated in the graph. Scale bar, 300 μm.

**Figure 6:**
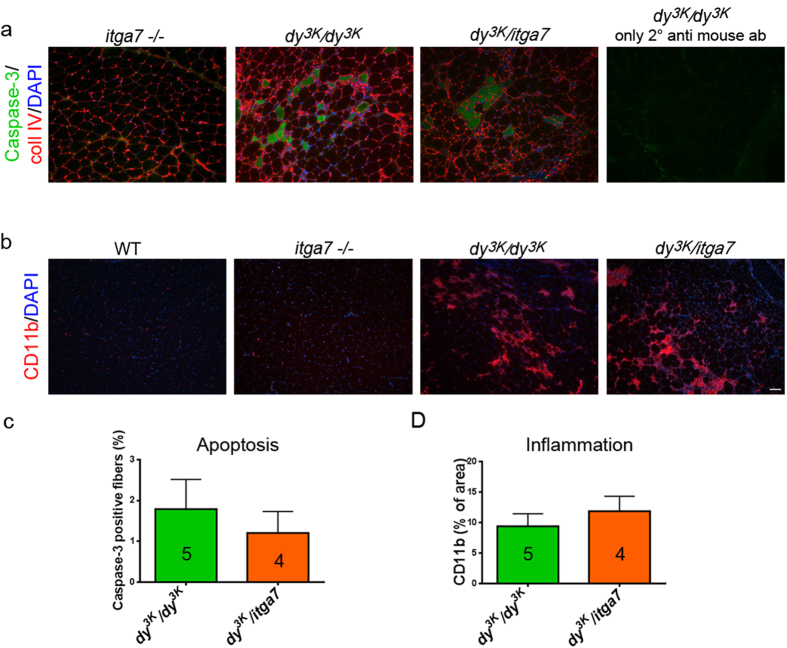
Muscles from *dy*^*3K*^*/dy*^*3K*^ and *dy*^*3K*^/*itga7* are equally affected with apoptosis and inflammation. (**a**) Caspase-3 immunostaining (green) reveals spread apoptotic fibers or group of dying cells in both *dy*^*3K*^*/dy*^*3K*^ and *dy*^*3K*^/*itga7* muscles. Collagen IV antibody (red staining) and DAPI (blue) were used to delineate muscle fibers and show nuclei. (**b**) CD11b staining (red) showed infiltration of macrophages into *dy*^*3K*^*/dy*^*3K*^ and *dy*^*3K*^/*itga7* dystrophic triceps muscle. DAPI (blue) depicts cell nuclei. (**c**) Percentage of apoptotic fibers was not increased in double knockout triceps muscle compared to *dy*^*3K*^*/dy*^*3K*^ triceps (p = 0.6828, Mann-Whitney test). (**d**) Amount of macrophages was not significantly different between *dy*^*3K*^*/dy*^*3K*^ and *dy*^*3K*^/*itga7* triceps (p = 0.7143, Mann-Whitney test). The numbers of animals used are indicated in the graph. Scale bar, 50 μm.

Although the dystrophic phenotype of muscles from double knockout mice is not drastically worsened compared to *dy*^*3K*^*/dy*^*3K*^ muscles (Fig. 3a), slightly impaired/slower regeneration (Fig. 3b) could result in more rapid loss of muscle fibers and in consequence, give rise to increased fibrotic build-up. Thus, we analysed the expression of collagen III and fibronectin, two major components of fibrotic lesions, in triceps and quadriceps muscle (Fig. 5). Integrin α7-deficient muscles did not exhibit fibrotic changes, whereas *dy*^*3K*^*/dy*^*3K*^and *dy*^*3K*^/*itga7* muscles showed increased collagen III and fibronectin production (Fig. 5a). The quantitative comparison of areas affected with collagen III and fibronectin deposition in *dy*^*3K*^*/dy*^*3K*^ and *dy*^*3K*^/*itga7* muscles (triceps and quadriceps) did not demonstrate a significant difference in fibronectin and collagen content between the genotypes (Fig. 5b). Yet, a trend for marginally enhanced fibrotic tissue build-up in double knockout triceps was observed (Fig. 5b, p = 0.0635), which could indeed be matched with moderate regeneration impairment in *dy*^*3K*^/*itga7* muscles. Nevertheless, it does not change the fact that muscles from both laminin α2 chain-null mice and double knockout mice are severely dystrophic and do not differ substantially between each other. This was also confirmed by analyses of apoptosis and inflammation (Fig. 6). Apoptosis is a hallmark of laminin α2 chain-deficient muscle and integrin α7β1 signalling is involved in maintenance of muscle survival^6^,^10^,^16^,^20^. We analysed apoptosis in both single and double knockout muscles (Fig. 6a). No increase in apoptosis was detected in double knockout muscles compared to *dy*^*3K*^*/dy*^*3K*^ muscles as indicated by caspase-3 immunostaining and quantification of caspase-3 positive fibers (p = 0.6828) (Fig. 6a,c). Integrin α7-deficient mice did not display apoptotic muscle cells (Fig. 6a).

Since inflammation is a feature of laminin α2 chain-deficient muscular dystrophy^12^,^44^,^45^,^46^, we assessed the inflammatory response in muscles from all three mouse models used in this study (Fig. 6b). CD11b immunostaining depicting macrophages revealed equally strong inflammatory response in both *dy*^*3K*^*/dy*^*3K*^ and *dy*^*3K*^/*itga7* muscles (p = 0.7143) (Fig. 6b,d) and showed almost no inflammation in *itga7*-null muscles (Fig. 6b).

## Discussion

Although numerous studies with muscular dystrophy patients and with genetically modified dystrophic animals have been performed, the molecular puzzle of relationships between different adhesion complexes in skeletal muscle has not been entirely deciphered. Laminin α2 chain is a bridging element between two adhesion complexes – integrin α7β1 and the DGC. The DGC remains intact in laminin α2 chain-deficient muscle and we have recently demonstrated non-redundant functions of laminin α2 chain and the DGC components dystrophin and β-sarcoglycan, as loss of these molecules significantly exacerbated the phenotype of *dy*^*3K*^*/dy*^*3K*^ mice^12^. Integrin α7 chain expression, on the other hand, is secondarily reduced in MDC1A^20,36,37^ and therefore we reasoned that mice deficient in both laminin α2 chain and integrin α7 chain should have a similar phenotype as laminin α2 chain-null mice^12^. In the current study we test this hypothesis by deleting the integrin α7 subunit in laminin α2 chain-deficient *dy*^*3K*^*/dy*^*3K*^ mice.

In general, the phenotype of double knockout mice did not exceed the severity of laminin α2 chain single knockout mice, which represent one of the most severe muscular dystrophy mouse models^12^. The fact that mice deficient in both laminin α2 chain and β-sarcoglycan or dystrophin (*dy*^*3K*^*/Sgcb* and *dy^3K^/mdx*, respectively) exhibited a dramatically deteriorated dystrophic phenotype^12^ indicates that integrin α7β1-laminin-211 axis could have an inferior role to the DGC-laminin-211 association in skeletal muscle. On the other hand, mice lacking integrin α7 subunit and the DGC components showed a very severe phenotype^32^,^33^,^34^ (albeit less severe than *dy*^*3K*^/*mdx* and *dy*^*3K*^/*Sgcb* mice^12^), implying that integrin α7β1 also contributes to the functional integrity of skeletal muscle. Consequently, we have demonstrated that truncated laminin that cannot bind dystroglycan but binds integrin α7β1 is sufficient to adequately maintain muscle function^35^. It could be that laminin α2 chain binding to integrin α7β1 has a more important role in signalling than in providing structural support to muscle cells^5^. However, the signalling cascades involving integrin α7β1 that are fundamental for muscle function are yet to be identified and this remains an important task.

We also sought to elucidate if compensatory upregulation of other α integrin subunits could be an alternative reason behind the not substantially aggravated phenotype of double knockout mice. Overlapping functions of different β1 integrins have been indicated in numerous studies^15^. Accordingly, integrin β1 subunit is not absent from cell membranes in laminin α2 chain-null and integrin α7/laminin α2 chain-deficient muscle (Fig. 1 and^37^). However, the undistinguishable expression pattern of integrin α6 and integrin α5 subunits in *dy*^*3K*^*/dy*^*3K*^ and *dy*^*3K*^/*itga7* muscles excludes the possibility that integrin α6β1 and/or integrin α5β1 prevent further deterioration of dystrophic phenotype in double knockout animals, even if integrin α5 subunit has been shown to protect muscle from damage^40^. What is more, neither of those integrin complexes seems to involved in inhibiting the dystrophic phenotype of single knockout *dy*^*3K*^*/dy*^*3K*^ mice. Contrary, the upregulation of integrin α5 chain, a major fibronectin receptor expressed in fibroblasts, has been shown to impact various fibrotic conditions^47^,^48^,^49^,^50^,^51^ and inflammation^52^,^53^. It is not excluded that upregulation of integrin α5 in laminin α2 chain-deficient muscles promotes fibrosis, as integrin α5 subunit does not bind laminin-211, but interacts with fibronectin.

Although the phenotype of double knockout *dy*^*3K*^/*itga7* muscles is to large extent the same as the phenotype of *dy*^*3K*^*/dy*^*3K*^ muscles, a more detailed analysis revealed a slight exacerbation of muscle defects, such as somewhat impaired regeneration and tendency for marginally increased fibrosis. These results match the data published by Doe *et al.*, where the overexpression of integrin α7 subunit has been shown to moderately improve the phenotype of *dy*^*W*^*/dy*^*W*^ mice^38^. On the other hand, reduced inflammation has also been suggested to be controlled by integrin α7 overexpression in *dy*^*W*^*/dy*^*W*^ muscle^38^. Yet, the deletion of integrin α7 subunit in *dy*^*3K*^*/dy*^*3K*^ mice did not exacerbate the inflammatory response, pointing toward no significant role for integrin α7β1 in inflammation regulation, at least in *dy*^*3K*^*/dy*^*3K*^ muscle. Collectively, results obtained in Burkin’s laboratory and our data suggest that the upregulation of integrin α7 subunit in laminin α2 chain-deficiency could only affect the muscle phenotype to some extent, most likely influencing muscle regeneration and perhaps muscle survival^21^. Indeed, there is a line of evidence for the role of integrin α7β1 in muscle regeneration and satellite cell activation/myogenic function^22^,^31^,^33^,^54^,^55^. Besides MDC1A, the integrin α7β1 upregulation approach has also been tested for Duchenne muscular dystrophy. Both transgenic overexpression and AVV-mediated delivery of integrin α7β1 have shown rather good effects in dystrophin/utrophin-deficient mice (*mdx/utr*) and dystrophin-null mice (*mdx*), respectively^30^,^31^,^56^,^57^. Thus, targeting integrin α7β1 expression may show more promise for treatment of Duchenne muscular dystrophy.

In summary, our previous results indicate that DGC and laminin α2 chain have complementary but non-redundant functions in skeletal muscle while the current study shows that integrin α7β1 does not play any other roles in skeletal muscle than mediating the laminin α2 chain interaction. Moreover, integrin α7β1 probably does not bind any additional vital ligands. Laminin α2 chain, on the other hand, may interact with other receptors than dystroglycan and integrin α7β1. Potential receptors may include integrin α9β1, which is expressed in skeletal muscle^58^ and has been shown to bind laminin-111^59^. Also, proteins that were originally identified in Schwann cells as laminin-binding molecules (galactosyl-sulfatides^60^ and adhesion G protein-coupled receptors^61^) may serve as laminin α2 chain binding receptors in skeletal muscle. In order to further understand the molecular pathology of MDC1A and other muscular dystrophies it will be crucial to validate whether laminin-211 interacts with additional receptors in striated muscle.

## Methods

### Mouse models, double knockout mice generation

Laminin α2 chain-null *dy*^*3K*^*/dy*^*3K*^ mice were previously described^10^. *Itga7*/+ mice (B6;129-*Itga7*^*tm1Burk*^/J)^42^ were obtained from Jackson laboratory. *Dy*^*3K*^*/*+ males or females were bred with *itga7*/+ females or males, respectively. The resulting *dy*^*3K*^*/*+; *itga7*/+ mice were mated to generate wild-type, *itga7* knockout, *dy*^*3K*^*/dy*^*3K*^and *dy*^*3K*^/*itga7* double knockout mice. Control animals were: wild-type or *dy*^*3K*^/+. Mice were maintained in the animal facilities of Biomedical Center (Lund) according to the animal care guidelines. All experimental procedures involving animals were approved by the Malmö/Lund (Sweden) Ethical Committee for Animal Research (the ethical permit number: M15-12 and M152-14) in accordance with the guidelines approved by the Swedish Board of Agriculture.

### Histology and morphometric analysis

Quadriceps femoris, triceps brachii, diaphragm and heart muscles were isolated from 5-7-week-old mice (single knockouts, double knockouts and control animals), embedded in OCT and frozen rapidly in liquid nitrogen. Cryosections (7 μm) were stained with hematoxylin and eosin^12^ or picrosirius red/fast green^44^. Stained cross-sections were scanned using Aperio’s Scanscope CS2 (with Scanscope console v.8.2.0.1263) and images were created using Aperio software.

Centrally nucleated fibers representing regenerating muscle cells and peripherally nucleated normal muscle cells were counted in triceps brachii and vastus intermedius (quadriceps femoris) from *dy*^*3K*^*/dy*^*3K*^ and *dy*^*3K*^/*itga7* mice using ImageJ software version 143u (NIH, Bethesda, MD). A whole area of each muscle cross section (from both legs) was considered. Percentage of centrally nucleated fibers in each muscle was calculated and averaged between two collateral muscles from the same animal. Mann-Whitney test was used for statistical analysis (p < 0.05).

### Immunofluorescence

Cryosections were subjected to immunofluorescence labeling^12^ with antibodies against: laminin α2 chain (rat monoclonal 4H8-2, 1:100, Alexis Biochemicals), integrin α7B subunit (rabbit polyclonal U31, 1:300, kindly provided by Dr. U. Mayer), integrin β1D subunit (mouse monoclonal 2B1, 1:80, Millipore), integrin α5 subunit (rat monoclonal 5H10-27 phycoerythrin-conjugated, 1:100, Abcam), integrin α6 subunit (rat monoclonal GoH3, 1:200, Abcam), CD11b (rat monoclonal M1/70, 1:250, BD Pharmingen), collagen III (goat polyclonal, 1:100, Southern Biotech), fibronectin (rabbit polyclonal, 1:1000, Abcam), embryonic myosin heavy chain (mouse monoclonal F1.652, 1:10; Developmental Studies Hybridoma Bank), caspase-3 (mouse monoclonal 46, 1:100, BD Transduction Laboratories), collagen IV (rabbit polyclonal, 1:100, Millipore), dystrophin (rabbit polyclonal, 1:100, Abcam), laminin γ1, laminin α4 and laminin α5 chain (all rabbit polyclonal, 1:100, kindly provided by Dr. T. Sasaki). Primary antibodies were detected with proper secondary antibodies (Molecular Probes). Stained cross-sections were analysed using a Zeiss Axioplan fluorescence microscope. Images were taken using with an ORCA 1394 ER digital camera and Openlab 4 software, at the same exposure times for all genotypes. The area corresponding to CD11b, collagen III and fibronectin labeling was quantified in relation to the entire area of vastus intermedius (quadriceps femoris) and/or triceps brachii cross-sections from *dy*^*3K*^*/dy*^*3K*^ and *dy*^*3K*^/*itga7* mice (for each animal the average percentage of stained area was calculated for muscles from collateral legs). ImageJ software version 143u (NIH, Bethesda, MD) was used. Mann-Whitney test was used for statistical analysis (p<0.05). For collagen III and fibronectin staining, myotendinous junctions were excluded from quantification.

Caspase-3 positive fibers and normal muscle fibers were counted in triceps from *dy*^*3K*^*/dy*^*3K*^ and *dy*^*3K*^/*itga7* mice using ImageJ software version 143u (NIH, Bethesda, MD). Percentage of caspase-3 positive fibers was calculated. Mann-Whitney test was used for statistical analysis (p < 0.05).

### Statistical analysis

All statistical analyses were performed with GraphPad Prism software version 6 (La Jolla, CA). Averaged data were reported as means ± SEM. Mann-Whitney test was used. Statistical significance was accepted for p < 0.05.

## Additional Information

**How to cite this article**: Gawlik, K. I. and Durbeej, M. Deletion of integrin α7 subunit does not aggravate the phenotype of laminin α2 chain-deficient mice. *Sci. Rep.* **5**, 13916; doi: 10.1038/srep13916 (2015).

## Supplementary Material

Supplementary Information
