# Supplementary material for: Deletion of integrin α7 subunit does not aggravate the phenotype of laminin α2 chain-deficient mice
Source: Sci Rep. 2015 Sep 10;5:13916. doi: 10.1038/srep13916 (PMC4564817; doi:10.1038/srep13916)
Supplement: Supplementary Information [file srep13916-s1.doc]

**Deletion of integrin α7 subunit does not aggravate the phenotype of laminin α2 chain-deficient mice**

**Kinga I. Gawlik^1^* and Madeleine Durbeej^1^**

^1^Department of Experimental Medical Science, Muscle Biology Unit, Lund University, Sweden

*Corresponding Author:

Kinga I. Gawlik

Muscle Biology Unit

Department of Experimental Medical Science

BMC C12, Lund University

221 84 Lund, Sweden

Tel : 0046 46 2220813

Fax: 0046 46 2220855

Email: [kinga.gawlik@med.lu.se](mailto:kinga.gawlik@med.lu.se)

**
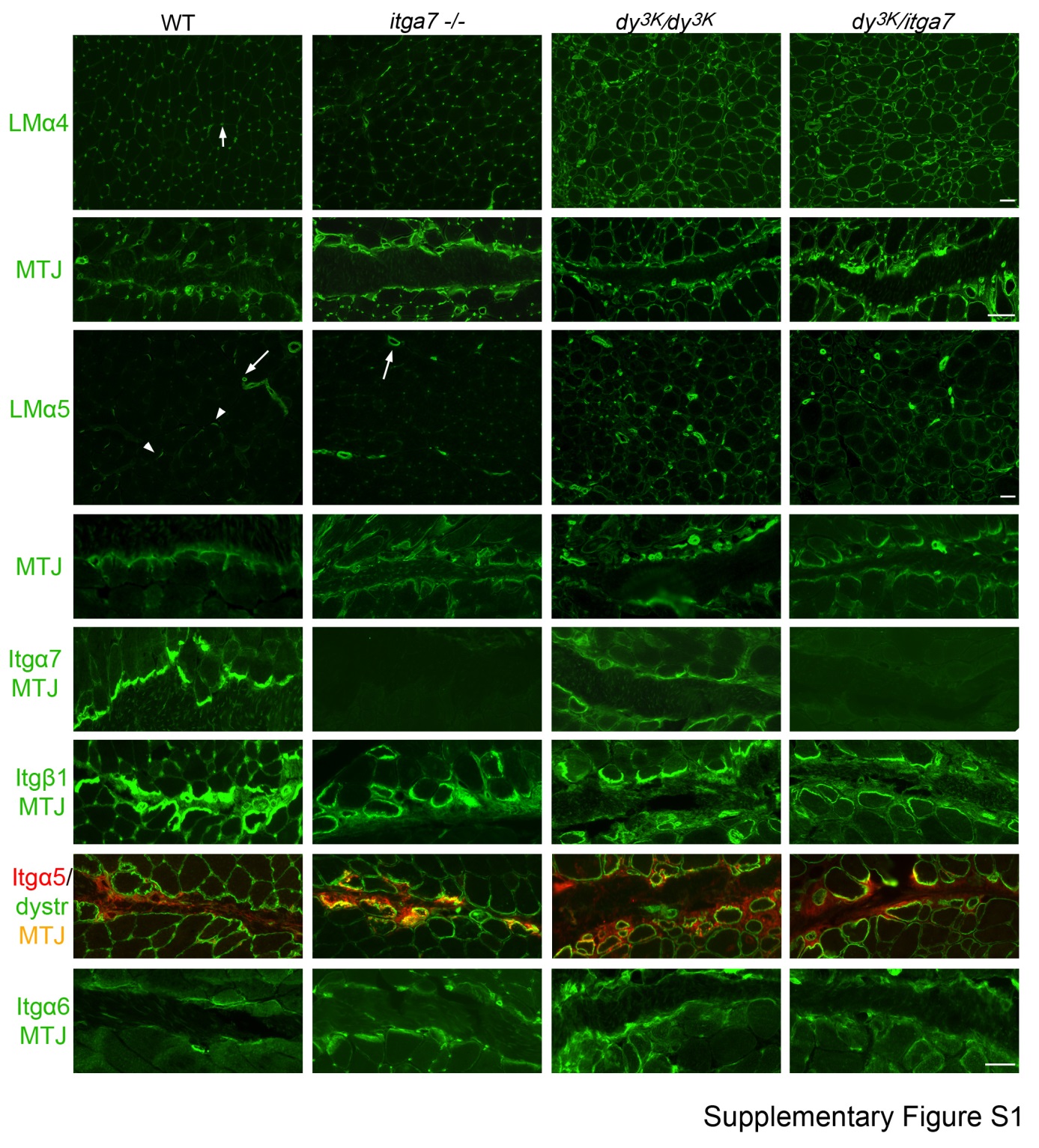
**

**Supplementary Figure 1.** Expression pattern analysis of laminin α4 and α5 as well as various integrin subunits (α7, α5, α6, β1) in muscle and in the myotendinous junction (MTJ) of wild-type, *itga7*-null, *dy^3K^/dy^3K^* and *dy^3K^*/*itga7* mice. Laminin α4 and α5 chains are expressed in blood vessels (white arrows), at the neuromuscular junction (white arrowheads) and at the MTJ in wild-type and *itga7* -/- mice. These laminin chains are upregulated in the extrasynaptic basement membranes in *dy^3K^/dy^3K^* and *dy^3K^*/*itga7* muscle. Additionally, laminin α4 chain is slightly upregulated at the MTJ in these muscles. Strong expression of integrin β1 (Itgβ1, green) has been maintained at the MTJ in single and double knockout muscles. Integrin α7 (Itgα7, green) expression was preserved in the *dy^3K^/dy^3K^* MTJ. Integrin α5 (Itgα5, red) is not expressed in the MTJ of wild-type mice (co-stained with dystrophin; green), but it is present in the MTJs in all three mutants analysed (at low levels in *dy^3K^/dy^3K^* and *dy^3K^*/*itga7* MTJs). Integrin α6 (Itgα6, green) is weakly expressed in the wild-type MTJ. It is slightly increased in the MTJs of *itga7* null, *dy^3K^/dy^3K^* and *dy^3K^*/*itga7* mice. Scale bars, 40 μm.
